# Supplementary material for: Development of a computer-aided design software for the quantitative evaluation of aesthetic damage
Source: PLoS One. 2019 Dec 18;14(12):e0226322. doi: 10.1371/journal.pone.0226322 (PMC6919621; doi:10.1371/journal.pone.0226322)
Supplement: S1 Code site — 1. (ZIP) [file pone.0226322.s001.zip › [EESC_jr][Projeto]Relatorio_07_21/reports/templates_zip/gentelella-master/production/index.html]

Gentellela Alela! | 


Gentellela Alela!

Welcome,

## John Doe

  


### General

- Home 
  - Dashboard
  - Dashboard2
  - Dashboard3
- Forms 
  - General Form
  - Advanced Components
  - Form Validation
  - Form Wizard
  - Form Upload
  - Form Buttons
- UI Elements 
  - General Elements
  - Media Gallery
  - Typography
  - Icons
  - Glyphicons
  - Widgets
  - Invoice
  - Inbox
  - Calendar
- Tables 
  - Tables
  - Table Dynamic
- Data Presentation 
  - Chart JS
  - Chart JS2
  - Moris JS
  - ECharts
  - Other Charts
- Layouts 
  - Fixed Sidebar
  - Fixed Footer

### Live On

- Additional Pages 
  - E-commerce
  - Projects
  - Project Detail
  - Contacts
  - Profile
- Extras 
  - 403 Error
  - 404 Error
  - 500 Error
  - Plain Page
  - Login Page
  - Pricing Tables
- Multilevel Menu 
  - Level One- Level One
      - Level Two
      - Level Two
      - Level Two
    - Level One
- Landing Page Coming Soon

- John Doe
  - Profile
  - 50%
    Settings
  - Help
  - Log Out
- 6
  - John Smith
    3 mins ago

    Film festivals used to be do-or-die moments for movie makers. They were where...
  - John Smith
    3 mins ago

    Film festivals used to be do-or-die moments for movie makers. They were where...
  - John Smith
    3 mins ago

    Film festivals used to be do-or-die moments for movie makers. They were where...
  - John Smith
    3 mins ago

    Film festivals used to be do-or-die moments for movie makers. They were where...
  - **See All Alerts**


Total Users

2500

*4%*  From last Week

Average Time

123.50

*3%*  From last Week

Total Males

2,500

*34%*  From last Week

Total Females

4,567

*12%*  From last Week

Total Collections

2,315

*34%*  From last Week

Total Connections

7,325

*34%*  From last Week

### Network Activities Graph title sub-title

December 30, 2014 - January 28, 2015

## Top Campaign Performance

Facebook Campaign

Twitter Campaign

Conventional Media

Bill boards

  

## App Versions

- - Settings 1
  - Settings 2

#### App Usage across versions

0.1.5.2

60% Complete

123k

0.1.5.3

60% Complete

53k

0.1.5.4

60% Complete

23k

0.1.5.5

60% Complete

3k

0.1.5.6

60% Complete

1k

## Device Usage

- - Settings 1
  - Settings 2

| Top 5 | Device  Progress |
| --- | --- |
|  | IOS \| 30%; Android \| 10%; Blackberry \| 20%; Symbian \| 15%; Others \| 30% |

## Quick Settings

- - Settings 1
  - Settings 2

- Settings
- Subscription
- Auto Renewal
- Achievements
- Auto Renewal
- Achievements
- Logout

#### Profile Completion

$
3,200
$5,000

## Recent Activities Sessions

- - Settings 1
  - Settings 2

- ## Who Needs Sundance When You’ve Got Crowdfunding?

  13 hours ago by Jane Smith

  Film festivals used to be do-or-die moments for movie makers. They were where you met the producers that could fund your project, and if the buyers liked your flick, they’d pay to Fast-forward and… Read More
- ## Who Needs Sundance When You’ve Got Crowdfunding?

  13 hours ago by Jane Smith

  Film festivals used to be do-or-die moments for movie makers. They were where you met the producers that could fund your project, and if the buyers liked your flick, they’d pay to Fast-forward and… Read More
- ## Who Needs Sundance When You’ve Got Crowdfunding?

  13 hours ago by Jane Smith

  Film festivals used to be do-or-die moments for movie makers. They were where you met the producers that could fund your project, and if the buyers liked your flick, they’d pay to Fast-forward and… Read More
- ## Who Needs Sundance When You’ve Got Crowdfunding?

  13 hours ago by Jane Smith

  Film festivals used to be do-or-die moments for movie makers. They were where you met the producers that could fund your project, and if the buyers liked your flick, they’d pay to Fast-forward and… Read More

## Visitors location geo-presentation

- - Settings 1
  - Settings 2

## 125.7k Views from 60 countries

|  |  |
| --- | --- |
| United States | 33% |
| France | 27% |
| Germany | 16% |
| Spain | 11% |
| Britain | 10% |

## To Do List Sample tasks

- - Settings 1
  - Settings 2

- Schedule meeting with new client
- Create email address for new intern
- Have IT fix the network printer
- Copy backups to offsite location
- Food truck fixie locavors mcsweeney
- Food truck fixie locavors mcsweeney
- Create email address for new intern
- Have IT fix the network printer
- Copy backups to offsite location


## Daily active users Sessions

- - Settings 1
  - Settings 2

**Monday**, 07:30 AM
F
**C**

## Texas *Partly Cloudy Day*

### 23

## Mon

### 25

##### 15 *km/h*

## Tue

### 25

##### 12 *km/h*

## Wed

### 27

##### 14 *km/h*

## Thu

### 28

##### 15 *km/h*

## Fri

### 28

##### 11 *km/h*

## Sat

### 26

##### 10 *km/h*


Gentelella - Bootstrap Admin Template by Colorlib
